# Hyperspectral Remote Sensing for Phenotyping the Physiological Drought Response of Common and Tepary Bean

**DOI:** 10.34133/plantphenomics.0021

**Published:** 2023-01-16

**Authors:** Christopher YS Wong, Matthew E Gilbert, Marshall A Pierce, Travis A Parker, Antonia Palkovic, Paul Gepts, Troy S Magney, Thomas N Buckley

**Affiliations:** Department of Plant Sciences, University of California, Davis, Davis, CA 95616, USA.

## Abstract

Proximal remote sensing offers a powerful tool for high-throughput phenotyping of plants for assessing stress response. Bean plants, an important legume for human consumption, are often grown in regions with limited rainfall and irrigation and are therefore bred to further enhance drought tolerance. We assessed physiological (stomatal conductance and predawn and midday leaf water potential) and ground- and tower-based hyperspectral remote sensing (400 to 2,400 nm and 400 to 900 nm, respectively) measurements to evaluate drought response in 12 common bean and 4 tepary bean genotypes across 3 field campaigns (1 predrought and 2 post-drought). Hyperspectral data in partial least squares regression models predicted these physiological traits (*R*^2^ = 0.20 to 0.55; root mean square percent error 16% to 31%). Furthermore, ground-based partial least squares regression models successfully ranked genotypic drought responses similar to the physiologically based ranks. This study demonstrates applications of high-resolution hyperspectral remote sensing for predicting plant traits and phenotyping drought response across genotypes for vegetation monitoring and breeding population screening.

## Introduction

Climate adaptation necessitates breeding for crop varieties with better yields, stress tolerance, and water-use efficiency [1,2]. Therefore, high-throughput phenotyping tools are needed to cost effectively and quickly screen physiological and biochemical characteristics across genotypes and environmental conditions [3–6]. Physiological variables such as stomatal conductance and leaf water potential (LWP) are indicators of plant water status and often used for evaluating drought tolerance [7,8]. However, monitoring plant water status requires extensive physical labor (i.e., personnel and time) and can be both subjective and destructive. Thus, remote sensing tools are being explored for objective high-throughput phenotyping applications.

Proximal remote sensing offers a powerful tool for high-throughput phenotyping of plant physiology [9–11]. Remote sensing techniques such as thermal-based [12,13], lidar-based [14,15], and optical-based [16] methods have shown promise for assessing plant water status. Sensors can be deployed on various platforms that include handheld instruments, ground-based vehicles, towers, unoccupied aerial vehicles, piloted aircraft, and satellites, all with different spatial and temporal trade-offs [4]. Hyperspectral data offer the most flexibility for assessing an array of physiological variables and structural plant traits, whereas thermal and lidar data are more narrowly suited for assessing evapotranspiration and canopy structure, respectively [17–19]. The advantage of hyperspectral reflectance data is their sensitivity to variation in pigments, water content, and leaf and canopy structure [20–22]. Thus, hyperspectral data may be used to remotely capture variation in plant physiology and structure across a range of timescales.

Traditionally, simple remotely sensed vegetation indices exploit variations in specific spectral bands to act as proxies of canopy structure and functions such as absorbed photosynthetically active radiation, leaf area index, and photosynthetic activity [23,24]. Beyond simple vegetation indices, full range visible (VIS)–near infrared (NIR) hyperspectral data enable machine-learning techniques such as partial least squares regression (PLSR) [25,26] to predict specific biochemical and physiological traits [27–32]. These models adjust weights applied to each spectral region to optimize model calibration for each trait. For example, the VIS region (400 to 700 nm) is sensitive to chlorophyll and carotenoid pigments and is often used as a proxy of photosynthetic activity [22]; the red edge (680 to 740 nm) is sensitive to chlorophyll content [33]; the NIR (740 to 900 nm) is sensitive to canopy structure and phenology [34]; and the shortwave near infrared (SWIR; 900 to 2,400 nm) is sensitive to water content and foliar biochemistry such as nitrogen and cellulose [35,36]. Therefore, with proper PLSR model calibration, remotely sensed hyperspectral data may be optimized using specific spectral regions to assess plant water status across genotypes.

The objective of this study was to utilize hyperspectral data from a ground-based handheld instrument and a tower-based system for PLSR modeling of 2 physiological traits—stomatal conductance and LWP—across a diverse array of beans (*n* = 16 genotypes; 12 common beans [*Phaseolus vulgaris* L.] and 4 tepary beans [*Phaseolus acutifolius* A. Gray]). The common bean genotypes represent populations varying in drought tolerance from different origins including eco-geographic race Mesoamerica, dry highland adapted accessions, and arid northern Mexican highland accessions [37]. Tepary beans, native to semiarid and arid environments, are generally more drought tolerant than common beans [38]. We explore these common and tepary bean genotypes in a field experiment with irrigated (control) and terminal drought treatments. The PLSR models were calibrated using hyperspectral data in the visible and NIR from 400 to 900 nm (tower- and ground-based) and full range hyperspectral data in the visible, NIR, and SWIR from 400 to 2,400 nm (ground-based) to explore the impacts of spatial scaling and spectral range on PLSR model performance. Finally, we apply the PLSR model predictions to phenotype and identify genotypic variation in drought response.

## Methods

### Study site

The experimental design consisted of a diverse multiparent breeding population of 300 common bean (*P. vulgaris* L.) [37] and 20 tepary bean (*P. acutifolius* A. Gray) genotypes, the latter generally being considered more drought tolerant [38]. Each genotype was represented by 3 randomly located replicate plots for a total of 960 plots per treatment. From the 320 genotypes, a subset of 16 genotypes (12 common and 4 tepary beans) were selected for intensive direct physiological measurements using traditional techniques (i.e., stomatal conductance and LWP), resulting in 96 measured plots across genotypes, replicates, and treatments. The common bean genotypes included the 8 parents of a multiparent (MAGIC) population and 4 progeny lines in this population. The 8 parents were chosen based on prior whole-plant phenotypic evidence of drought tolerance [37]. They represented a broad diversity of the Mesoamerican domesticated gene pool, including representation of the 3 major eco-geographic races [39].

All genotypes were grown in the field at the Plant Sciences Field Facility of the University of California, Davis (38.534°N, 121.775°W) from 2021 June to October in designated irrigated (control) and terminal drought treatments with 3 replicate plots per genotype. Seeds were planted on June 4. Each plot was 3.05 m long (N–S) and 1.52 m wide (E–W) with 2 planted rows spaced 66 cm apart separated from adjacent plots by unplanted rows (bare soil) 1.22 m long (N–S) or 1.52 m wide (E–W). Planting included a buffer row along the border of the field to account for border effects. During initial growth, both treatments were watered using aboveground drip irrigation, then switched to subsurface drip (50 cm deep) irrigation after stand establishment. We applied terminal drought by stopping irrigation to the drought treatments on July 26. To dry down the plants for harvest, irrigation was also terminated for the control plots on September 1.

Ground-based physiological measurements (stomatal conductance, predawn and midday LWP, ground-based hyperspectral, and soil moisture) were collected during 3 field campaigns. Campaign 1 occurred from July 5 to July 9 to provide a baseline comparison before ceasing irrigation to the drought plots; Campaign 2 occurred 2 weeks after terminal drought was initially imposed, from August 9 to August 13; and Campaign 3 occurred 4 weeks after terminal drought, from August 23 to August 26.

### Soil moisture neutron probe

In each plot of the 16 genotypes, we installed 1.5-m-long access tubes, made from galvanized steel electrical conduit with plastic end caps at the bottom end, into the ground prior to germination. We assessed soil moisture at 20-, 50-, 80-, 100-, 120-, and 140-cm depths using a neutron backscatter detector soil moisture probe (503 ELITE Hydroprobe, InstroTek Inc., Raleigh, NC, USA). We measured the baseline standard count above ground level at 1 m. Then, the neutron probe was inserted into the access tube to each depth and neutron counts were recorded. We determined relative soil moisture by calculating the ratio between soil counts and standard count; higher ratios indicated higher soil moisture contents. For each campaign, measurements were completed in 1 day between 10 and 13 h.

### Stomatal conductance

We measured leaf stomatal conductance (*g*_s_) on one leaf per plant for 5 to 8 plants per plot, at 8, 10, 12 and 14 h (measuring approximately one-fifth of all plots per day in each 5-day campaign), using a Delta-T AP4 porometer (Delta-T Devices Ltd, London, UK), calibrated before each measurement cycle.

### Leaf water potential

We measured LWP for 2 leaves per plot, collected at predawn (within 30 min prior to sunrise) and midday (between 13 and 14 h) using a Scholander pressure chamber (PMS Instrument Company, Albany, OR, USA). Within 2 s of excision with sharp secateurs, each leaf was placed in a Ziploc bag (which had previously been breathed into for humidification), and the bag was flattened to remove excess air and then sealed and immediately enclosed in a cooler filled with ice, followed by transport to the laboratory for measurement within 3 h.

### Drone imagery

Unoccupied aerial vehicles (or drones) were used to systematically measure canopy volume, normalized difference vegetation index (NDVI), and canopy temperature across all field plots during each field campaign. Drone methods and hardware were modified from Parker et al. [40]. Using the difference between canopy digital surface models and soil digital surface models, and vegetation canopy area and height, canopy volume was estimated [40]. A Micasense RedEdge-M multispectral camera (now AgEagle Aerial Systems Inc., Wichita, KS, USA) and a Zenmuse XT-R thermal camera were mounted onto a DJI Matrice 100 (DJI Inc., Shenzhen, China) to collect field imagery. Flight plans for data collection were programmed and uploaded to the aircraft using DJI Ground Station Pro. Flights were conducted at an altitude of 30 m for volume and NDVI measurements, and 60 m for thermal imagery. A minimum of 80% front and side overlap was used between images, which were processed into field-scale orthomosaics using Pix4Dmapper Pro v4.6.4. Data extraction was done using the Create Grid and Raster Layer Zonal Statistics functions of QGIS v3.10.14.

### Ground-based hyperspectral reflectance

A ground-based handheld instrument (HR-1024i, Spectra Vista Corporation, Poughkeepsie, NY, USA) was used to measure reflectance spectra from 400 to 2,400 nm (3.3 to 9.5 nm full width at half maximum) at every subset plot from 11 to 13 h, once per week from July 12 to October 4, beginning 1 week after Campaign 1 and continuing through and beyond Campaigns 2 and 3. Measurement dates were all completed under clear sunny sky conditions. We used a fiber optic with a 4° field of view to measure the top of the canopy from a distance of 1 m. Three representative reflected radiance measurements were obtained per plot. An irradiance measurement was made by pointing the foreoptic at a 99.9% reflective upward-facing white reference panel (Spectralon), and was made every ~15 plots (~10 min). To calculate reflectance, reflected radiance from vegetation was divided by the preceding white reference scan (irradiance). Spectral regions were filtered out from 990 to 1,020 nm due to instrument-specific hot pixels, and from 1,340 to 1,445 nm and 1,790 to 1,955 nm for atmospheric water absorption.

### Tower-based hyperspectral reflectance: PhenoSpec

A tower-based remote sensing system, PhenoSpec, was used to continuously monitor hyperspectral reflectance (400 to 900 nm; 1.34 nm full width at half maximum) from July 16 to October 15. The PhenoSpec is described in detail in Wong et al. (in review). This system was set up with a 10-m-tall tower located approximately in the middle of the field. We limited the viewing radius of the system to 72.7 m (250 ft) to maintain sufficient viewing angles for optical data quality and spatial coverage. This enabled the measurement of 672 plot targets (336 per treatment) with 178 genotypes represented in both treatments, including 27 of the 96 plots selected for intensive direct physiological measurements of stomatal conductance and LWP. At the top of the tower, PhenoSpec consists of an RGB (red, green, blue) camera (AXIS Q8685-E PTZ Network Camera, Axis Communications AB, Lund, Sweden) to target specific bean plots with a 360° pan, a ground-to-sky view from −45° to 90°, and 30× optical zoom. Mounted on top of the camera was an enclosed colocated 2D scanning telescope unit (Thorlabs Inc., NJ, USA) for simultaneous spectral reflectance measurements with a 0.7° field of view, spot targeting 12 to 86 cm diameter (depending on distance). The telescope was connected to a fiber optic cable extending to the base of the tower into a temperature-controlled enclosure that housed the spectrometer for hyperspectral radiance (FLAME, Ocean Insights, FL, USA). With a total of 723 scans (including both target and sky references), minimum 5 s each, a complete target cycle took about 3 h. Sky irradiance reference scans were acquired every 15 targets (within ~40 s of target scans), during which a diffuser (~12% transmission efficiency) was used to increase the field of view to 180°.

To calculate reflectance, target radiance was divided by the nearest-in-time sky irradiance reference scan (within ~40 s of target scans). To screen out plots that did not germinate well due to herbivory, we used the NDVI calculated using (R800 − R680) / (R800 + R680), where R800 and R680 are reflectances at 800 and 680 nm, respectively; NDVI is an indicator of greenness of the bean plots. An NDVI threshold of 0.8 was used during Campaign 2, to exclude poorly germinated plots containing soil background in the spectral signature from all analysis. Only midday spectra obtained from 11 to 15 h were used for analysis to minimize diurnal variation and sun-sensor geometry on the spectral signal (Wong et al., in review). Finally, reflectance spectra were averaged over 4 to 5 days from the duration of each campaign to ensure adequate representation of the plot spectral signature during the campaigns.

### Partial least squares regression

**Fig. 1.**
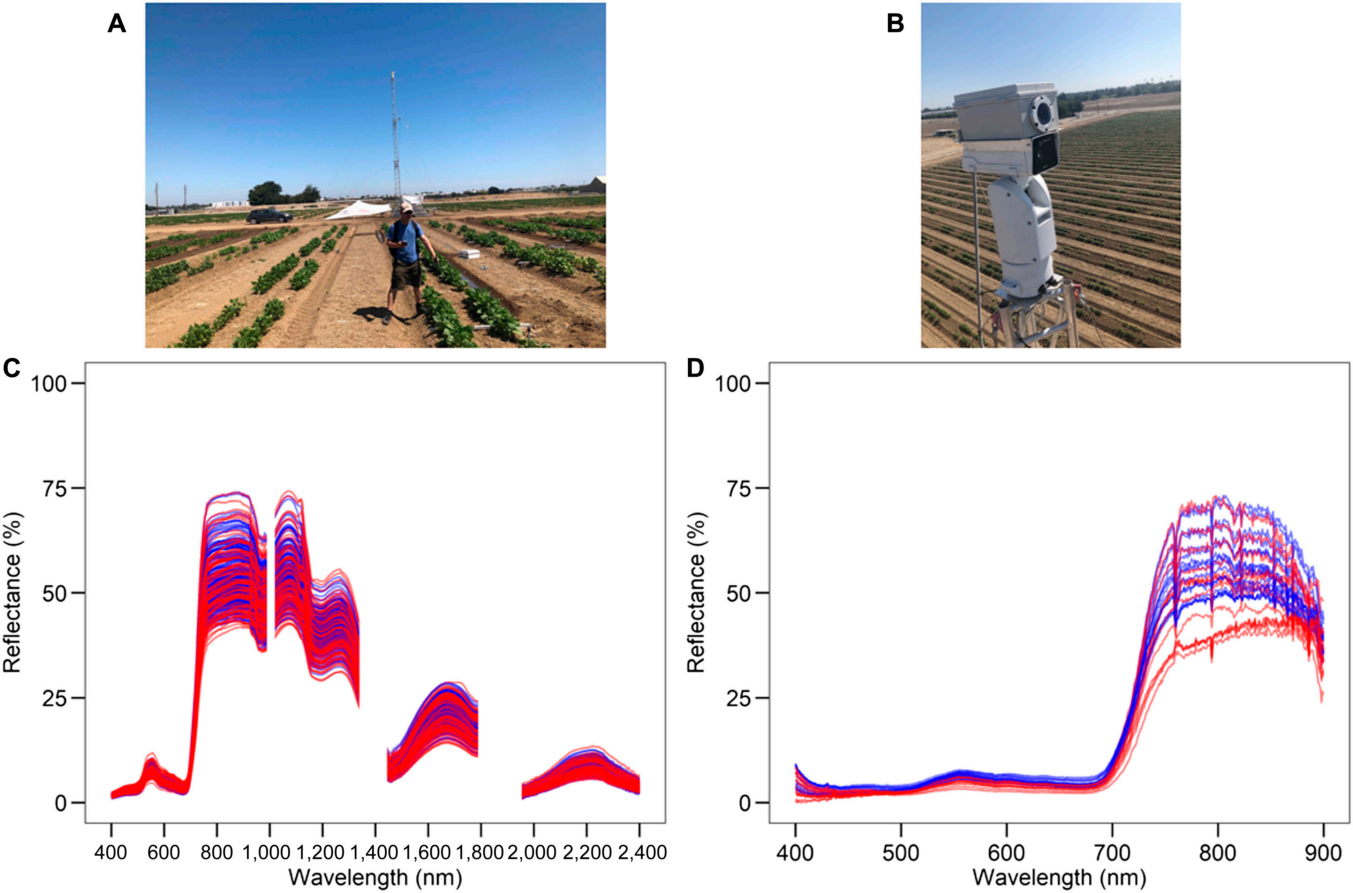
Hyperspectral reflectance of beans from (A and C) ground-based and (B and D) tower-based instruments for control (blue) and drought (red) treatments. Data shown are from the field campaigns; for the tower, only Campaigns 2 and 3 are shown. (For interpretation of the references to color in this figure legend, the reader is referred to the web version of this article.)

To predict midday stomatal conductance (from 12 h measurement cycle) and predawn and midday LWP, we used hyperspectral data from the ground-based handheld instrument and PhenoSpec (Fig. 1), henceforth referred to as ground and tower, respectively, with PLSR modeling. PLSR modeling was performed in R [41] using the “pls” package [42]. PLSR models were calibrated following the recommendations of Burnett et al. [43]. For ground-based PLSR, we used data from all 3 campaigns for model calibration (Fig. 1A and C). For tower-based PLSR, only Campaign 2 and 3 data were used (Fig. 1B and D), as plants were early in development during Campaign 1, resulting in soil background signals in the PhenoSpec spectra. With the ground-based instrument, we randomly split the data into model calibration and model validation 70/30% (*n* = 168 calibration and 72 validation points). For the tower-based instrument, due to limited sample size of validation plots within the tower field of view (*n* = 29 plots), data splitting was not possible, so all data were used for model calibration/validation. From the validation dataset, we determined the coefficient of determination (*R*^2^) and root mean square percent error (RMSPE) compared with ground-based physiological data for model performance evaluation. For the ground-based spectra, 2 PLSR models were used, one using the full range (400 to 2,400 nm; Ground_Fullrange_) and the other using a constrained range matching the tower-based instrument (400 to 900 nm; Ground_VISNIR_).

### Assessing genotypic drought response

To phenotype drought response across genotypes, we calculated the genotype mean from replicate plots per treatment and campaign. We then calculated relative percent difference as 100 * (D − C) / [(D + C) / 2], where D and C represent measurements from drought and control treatments, respectively, for a given genotype. Heatmaps were created using the observed values (stomatal conductance, LWP, drone-based NDVI, canopy temperature, and canopy volume) and PLSR predicted values from the Ground_VISNIR_ PLSR model, which was generally the best-performing. Tower-based PLSR models were not used due to limited sample size representing the subset genotypes (*n* = 29). Physiology and genotype clustering was completed per heatmap (campaigns, and observed- vs. PLSR-based).

## Results

### Physiological and structural response to drought

**Fig. 2.**
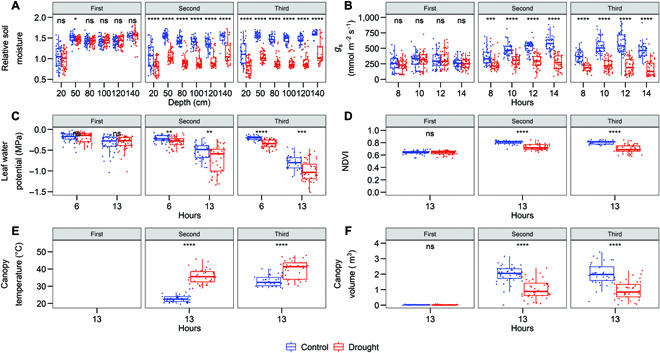
Boxplots showing the median and range across campaigns and treatments for (A) relative soil moisture, (B) stomatal conductance (*g*_s_), (C) predawn and midday leaf water potential (6 and 13 h refer to predawn and midday measurements, respectively), (D) drone-based NDVI, (E) canopy temperature, and (F) canopy volume. *P* value codes represent the *t* test significant mean difference (ns *P* > 0.05; *P < 0.05; **P < 0.01; ***P < 0.001; ****P < 0.0001) between control and drought treatments per campaign. Campaigns represent predrought baseline (First), and 2 and 4 weeks after terminal drought application (Second and Third, respectively). For genotype-specific variation, see Figs. S1 to S6.

Relative soil moisture, measured by the neutron backscatter probe, was similar between treatments and depths during Campaign 1, but differed between treatments and depths in Campaigns 2 and 3 (Fig. 2A). Stomatal conductance also differed between treatments in Campaigns 2 and 3, especially at 12 and 14 h, being lower in the drought treatment (Fig. 2B). Both predawn and midday LWPs were more negative in the drought treatment in Campaigns 2 and 3 (Fig. 2C); midday LWP also showed greater variation across genotypes within both treatments, compared to predawn LWP (Fig. 2C). Drone-based NDVI, canopy temperature, and canopy volume data revealed similar patterns, with no difference in Campaign 1 and treatment differences in Campaign 2 and 3 (Fig. 2D to F). Most parameters had larger variation in the drought treatment compared to the control in Campaigns 2 and 3.

### Hyperspectral PLSR predictions of plant traits

**Fig. 3.**
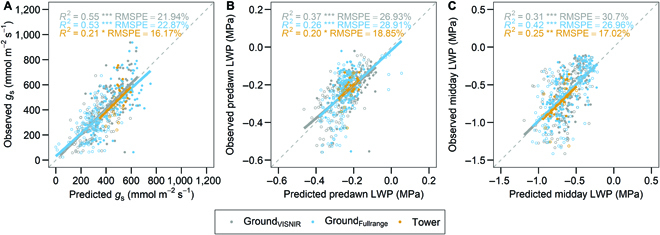
PLSR modeling of (A) midday stomatal conductance (*g*_s_), (B) predawn leaf water potential (LWP), and (C) midday LWP between Ground_VISNIR_ (gray), Ground_Fullrange_ (blue), and tower-based (yellow) PLSR models with respective coefficient of determination (*R*^2^) and root mean square percent error (RMSPE). Closed symbols represent control treatment and open symbols represent drought treatment plots. *P* value codes represent the *R*^2^ significance (ns *P* > 0.05; *P < 0.05; **P < 0.01; ***P < 0.001; ****P < 0.0001). (For interpretation of the references to color in this figure legend, the reader is referred to the web version of this article.)

PLSR predictions using hyperspectral reflectance best predicted stomatal conductance (*R*^2^ = 0.21 to 0.55; RMSPE 16 to 23%), followed by predawn LWP (*R*^2^ = 0.20 to 0.37; RMSPE 19 to 29%), and midday LWP (*R*^2^ = 0.25 to 0.42; RMSPE 17 to 31%) (Fig. 3). Compared to the tower, the ground-based method (Ground_VISNIR_ and Ground_Fullrange_) generally performed better for all 3 traits based on *R*^2^. There were notable differences in performance for both Ground_VISNIR_ and Ground_Fullrange_, depending on the predicted trait, suggesting that the full spectrum (400 to 2,400 nm) does not necessarily improve predictions. The Ground_VISNIR_ model performed better for predawn water potential (Fig. 3B), while the Ground_Fullrange_ model performed better for midday LWP (Fig. 3C). Both models performed similarly for stomatal conductance (Fig. 3A).

### Phenotyping drought response

**Fig. 4.**
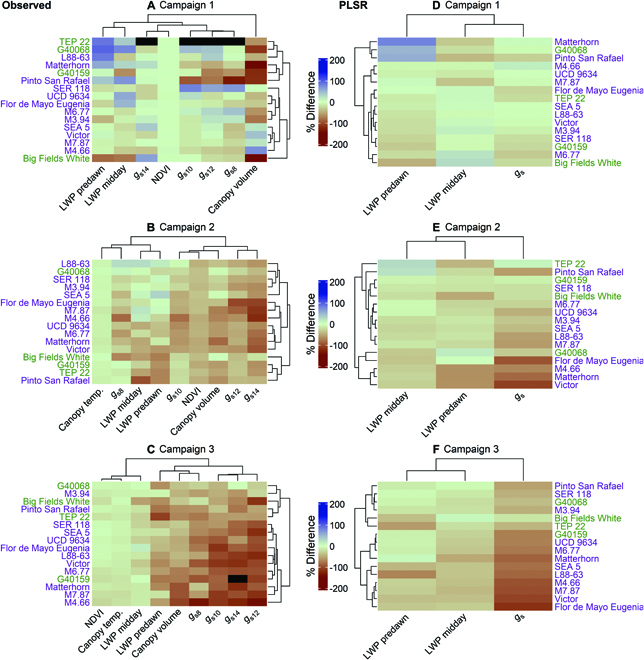
Heatmaps showing the relative percent difference of drought relative to control treatments across genotypes (purple: common bean; green: tepary bean), plant physiology, and field campaigns. (A) to (C) represent measured traits and (D) to (F) represent PLSR predicted traits from ground hyperspectral data. Parameter subscript for stomatal conductance (*g*_s_) represents hour of day. Black cells represent unavailable data. Canopy temperature relative % difference was multiplied by −1. (For interpretation of the references to color in this figure legend, the reader is referred to the web version of this article.)

Using heatmap clustering, we phenotype drought responses (drought relative to control treatments) for each genotype based on observed physiology (Fig. 4A to C) and PLSR predicted physiology (Fig. 4D to F). These heatmaps highlight drought response where more negative values represent larger reduction of drought treatment relative to the control treatment; thus, a relative percent difference closer to zero represents minimal drought response suggesting higher tolerance. For the observed physiology, similar genotype and physiology clustering is shown in Campaigns 2 and 3. Stomatal conductance, predawn LWP, and canopy volume showed the greatest genotypic variation in terms of relative percent difference. For Campaign 3, a cluster of genotypes near the top of the heatmap, where relative percent difference is close to zero, includes a combination of common (M3.94 and Pinto San Rafael) and tepary bean genotypes (G40068, TEP 22, and Big Fields White) (Fig. 4C). The PLSR model heatmaps highlight stomatal conductance as having the largest genotypic variation of relative percent difference followed by predawn LWP (Fig. 4E and F). The PLSR model for Campaign 3 shows similar clustering of genotypes, compared to the observed heatmap, where relative percent difference is closer to zero but includes additional common (L88-63 and SER 118) and tepary bean genotypes (G40158) (Fig. 4E and F).

## Discussion

In this study, we utilized remotely sensed hyperspectral reflectance data from ground- and tower-based instruments in PLSR models to assess plant water status and drought response across a subset of 16 diverse bean genotypes, varying in drought tolerance. We showed that ground-based PLSR models were effective in predicting stomatal conductance and predawn LWP. The tower-based models were limited by sample size (overlapping validation plots) but highlighted promise for up-scaling to a tower-based system for automated and continuous monitoring of hyperspectral data. Moreover, we phenotyped drought response using observed water status and PLSR predicted values and found similar clustering, suggesting that hyperspectral PLSR models are a promising high-throughput phenotyping tool for physiology-based selection in breeding programs.

### PLSR predictions of plant water status

Hyperspectral reflectance with PLSR modeling takes advantage of sensitive spectral regions for predicting an array of plant characteristics including photosynthetic parameters, pigment content, and leaf biochemistry across species and transgenic lines [28,30]. To phenotype drought response, we directly measured stomatal conductance and LWP, which are sensitive to plant water content and stress [44,45], and predicted these measurements using PLSR models driven by hyperspectral data. Our ground-based PLSR models predicted stomatal conductance well (Fig. 3A), similar to past studies in soybean and wheat [32,46 ,47]. Spectral regions of importance based on variable importance in projection (VIP) scores reveal that the green, red-edge, and NIR regions were important in model calibration (Fig. S7). These spectral regions are associated with variation of pigments (chlorophyll and carotenoids) and canopy structure [22,33 ,48]. While spectral variation is not directly sensitive to leaf gas exchange and therefore stomatal conductance, PLSR models exploit variation in chlorophyll pools, carotenoid composition, and structural effects such as leaf angle/wilting that may covary with stomatal conductance.

In contrast, remotely assessing LWP has historically proven challenging [49], as LWP arises from several disparate influences, including plant hydraulics, transpiration rate, and soil moisture [50]. Recent studies have demonstrated the potential of PLSR modeling for predicting LWP [51,52]. Our ground-based PLSR results support the potential of PLSR predictions of LWP, albeit with weaker performance when compared to stomatal conductance (Fig. 3). For predawn LWP, spectral data were acquired at a different time (noon). This decoupling may suggest that the spectral variation that predicted predawn LWP captures physiological properties that do not differ strongly between predawn and midday; alternatively, it may merely result from the biophysical correlation between predawn and midday water potentials. VIP scores for predawn LWP, while similar to those for stomatal conductance, show higher peaks in the green and lower peaks in NIR regions (Fig. S7), likely exploiting variation in pigment pools (chlorophyll and carotenoids) more than canopy structure compared to the stomatal conductance model. In contrast, midday LWP and spectral reflectance were acquired at similar times of day. Interestingly, this was the only variable for which the Ground_Fullrange_ PLSR model outperformed the Ground_VISNIR_ PLSR model. Here, VIP scores greatly favored the NIR regions, with the Ground_Fullrange_ PLSR model taking advantage of the SWIR regions, suggesting that PLSR models exploited variation in canopy structure and perhaps mesophyll structure [22,24]. We note that RMSPE was relatively high for midday LWP (27% to 31%). We suspect that model calibration was limited as midday LWP did not vary much across genotypes and treatments (Fig. 2C). This was likely due to high midday temperature (generally >35 °C in California's Central Valley) and low ambient vapor pressure causing high evaporative demand and low water potential in both control and drought treatment plants.

Comparing the different PLSR models between Ground_VISNIR_, Ground_Fullrange_, and Tower reveals the potential and limitations of different spectral regions and their upscaling potential (Fig. 3). Generally, the Ground_VISNIR_ PLSR model performed best (except for midday LWP, where the Ground_Fullrange_ PLSR model performed best) (Fig. 3C). This suggests that the benefit of SWIR in the PLSR model calibration is variable dependent [30] and that physiological variables more directly linked to water status will benefit from SWIR inclusion. Comparing the Ground_VISNIR_ PLSR model to the tower-based PLSR model highlights potential upscaling from observation distances of 1 m to 10 m. Unfortunately, the tower footprint with matching subset genotype plots was limited (*n* = 29), resulting in weaker model calibration. However, given the overlapping lines of best fit with the other models, the tower model shows promise for predicting plant traits in comparison to the ground-based instrument (Fig. 3), which could be further explored with a larger sample size of validation plots within the tower field of view. Interestingly, when comparing VIP scores, the ground-based models had higher VIP weights in the visible spectral regions, in contrast to the tower-based model which favored the NIR (Fig. S8). This is indicative of the sensitivity of the spectra across spatial scales; the tower-based PLSR model is likely more sensitive to canopy structure in the NIR [27], compared to the 1-m distance of the ground-based PLSR models capturing variation in chlorophyll and carotenoid dynamics in the visible region.

### Phenotyping genotypic drought response

Genotype-specific drought response was assessed using directly measured physiological and drone-based variables (Fig. 4). Stomatal conductance, predawn LWP, and canopy volume captured large genotypic variation in drought response relative to control treatments (Fig. 2). The heatmap clustering, largely based on these parameters, identified genotypic groupings for drought response. In Campaign 2, which represents the pod filling stage (R8: [53]), we identified 3 genotypic groupings (Fig. 4B). The first group, representing lower physiological drought response (i.e., smaller percent difference), included 3 eco-geographic race Mesoamerica (humid lowlands; [39]) common beans bred for drought tolerance (L88-63, SER 118, and SEA 5), a MAGIC population progeny (M3.94), and a tepary accession (G40068) [54,55]. The second group, representing larger physiological drought response (i.e., larger percent difference), consisted of 4 dry-highland-adapted accessions (eco-geographic race Durango; [39]) and 3 MAGIC progeny genotypes. Finally, the third group, also representing low physiological drought response, included 3 tepary beans (Big Fields White, G40159, and TEP 22) and a common bean cultivar (Pinto San Rafael) adapted to arid northern Mexican highlands [56]. In Campaign 3, which represents the maturation stage (R9: [53]), 2 main genotypic groupings were identified (Fig. 4C). The lower physiological drought response group included 3 tepary beans (G40068, Big Fields White, and TEP 22), the cultivar Pinto San Rafael, and the MAGIC progeny M3.94. The second group represented higher physiological drought response as indicated mainly by stomatal conductance, canopy volume, and predawn LWP. Overall, across the 2 post-drought campaigns, our results confirmed that tepary bean is generally more drought tolerant than common bean—sustaining higher gas exchange rates, greater predawn water potential, and canopy volume under drought. Compared to common beans, tepary bean is native to semiarid and arid environments, and is considered—on average—highly resistant to drought, relying on drought avoidance and tolerance mechanisms [38,57]. For example, tepary beans have fine root systems for soil penetration to access limited soil water reserves [58]. Interestingly, a few common bean lowland breeding lines (race Mesoamerica: SER 118, SEA 5, and M3.94) and the common bean cultivar (Pinto San Rafael) resemble tepary bean physiological responses, which supports breeding efforts for increased terminal drought tolerance in common and tepary bean. However, yield quantification will be needed to determine if physiological drought response represents a benefit or limitation to fitness under drought conditions; we were unable to measure bean yield in this study due to an early heavy rain event, which prevented field access and caused bean spoilage. Furthermore, the influence of genotypic variation in phenology (i.e., timing of maturity and flowering) should be further explored as phenology may influence part of the drought response. We attempted to minimize this influence by timing Campaigns 2 and 3 according to general pod filling and maturation stages, respectively.

Hyperspectral PLSR modeling of stomatal conductance and water potential with heatmap clustering identified similar genotypic groupings as the observed data (Fig. 4E and F). The lower physiological drought response group included tepary (G40068, Big Fields White, and TEP 22) and common (Pinto San Rafael, SER 118, and M3.94) bean genotypes, which matches the observed data heatmaps. Campaign 2 included additional common and tepary bean genotypes in the drought response groupings (Fig. 4E). This may be attributed to differences in drought stress severity between Campaigns 2 and 3, with the Campaign 2 exhibiting less spectral differentiation due to drought response influencing PLSR model performance. Overall, our results suggest that hyperspectral data and PLSR modeling may be used for phenotyping an array of traits for identifying drought-tolerant species and genotypes. Accuracy may be improved by incorporating additional traits associated with drought response and increasing sample size for improved model calibration. A balance between population size and ground validation sample size must be considered with available resources and acceptable error rates [59]. Tower-based remote sensing for high-throughput phenotyping may extrapolate to the full genotype population beyond our subset 16 from ground-based spectra, which could be explored in the future.

### Extrapolating PLSR predictions

**Fig. 5.**
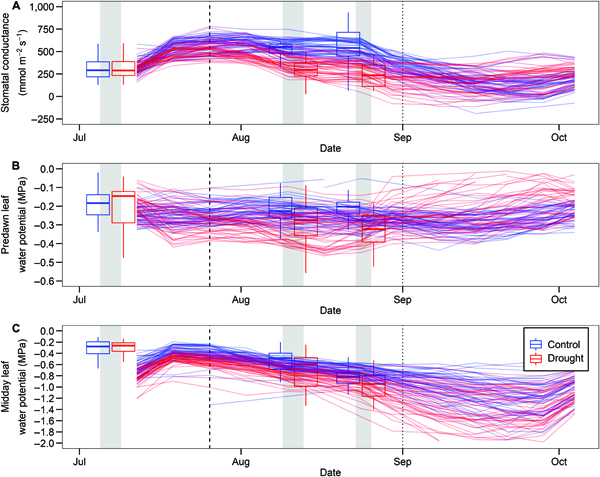
Extrapolating Ground_VISNIR_ PLSR models to the weekly timescale for (A) stomatal conductance, (B) predawn leaf water potential, and (C) midday leaf water potential. Lines represent plot-specific PLSR extrapolations; boxplot represents observed data from the field campaigns; gray bars represent Campaigns 1, 2, and 3 for ground truthing and model calibration, and vertical black lines represent the dates where irrigation was terminated for the drought (dashed) and control treatment plots (dotted).

A key benefit of remote sensing is its ability to screen a number of plots relatively quickly, therefore enabling data collection at higher temporal frequencies. Combined with intensive observation campaigns where physiological data are collected, remotely sensed PLSR models may be used to extrapolate beyond campaign dates and subset genotypes [60]. In this study, ground-based remote sensing was collected at the weekly timescale. In Fig. 5, we extrapolate the PLSR models to predict weekly stomatal conductance and water potential for the full growing season from mid-July to October. Stomatal conductance and predawn LWP show strong divergence between treatment plots starting in August after terminal drought (Fig. 5A and B). Midday leaf water showed less treatment divergence, following the observed data (Figs. 2C and 4C). Additionally, because the spectral data go well beyond the validation campaigns, much of the end-of-season data, which include some senescence, are beyond the means of our model calibration, and there is greater variability in predicted plant traits (Fig. 5). Therefore, Fig. 5 predictions should be taken as an example for future prospects of continuous (i.e., daily) remotely sensed monitoring of physiology with high uncertainty beyond September [61,62]. Interestingly, beyond September, with the PLSR stomatal conductance model, a decline was captured in the control treatment (after irrigation was terminated in the control plots), despite the PLSR model not being trained during these dates.

High-throughput phenotyping using hyperspectral remote sensing can provide a scalable, objective approach for quickly screening an array of plant traits over a diverse population, aiding in management strategies and breed selection. Our study highlights applications for remotely sensed hyperspectral data for assessing plant physiology related to water status in drought stress conditions to ultimately phenotype for drought response. By predicting multiple water status variables (stomatal conductance and water potential) with good accuracy using PLSR modeling, we identified potentially drought-tolerant common and tepary bean genotypes validated with in situ physiological measurements. An advantage of hyperspectral PLSR models is that they enable remotely sensed data to extrapolate predictions beyond the scope of direct measurements used to calibrate them, including to larger populations and beyond individual field campaigns. Hyperspectral PLSR models (and other machine learning techniques) can be calibrated across an array of plant traits, including biochemical and physiological traits [28,30], offering powerful remote sensing applications in precision agriculture and ecological monitoring.

## Data Availability

Data will be made available upon request.

## Supplementary Materials

Supplementary Materials Fig. S1. Genotype-specific relative soil moisture content at 20-, 40-, 80-, 120-, and 140-cm depths across 3 field campaigns. Fig. S2. Genotype-specific drought response of stomatal conductance from 8, 10, 12, and 14 h across 3 field campaigns. Fig. S3. Genotype-specific drought response of leaf water potential from predawn (6 h) and midday (13 h) across 3 field campaigns. Fig. S4. Genotype-specific drought response of midday drone-based normalized difference vegetation index across 3 field campaigns. Fig. S5. Genotype-specific drought response of midday drone-based canopy temperature across 3 field campaigns. Fig. S6. Genotype-specific drought response of midday drone-based canopy volume across 3 field campaigns. Fig. S7. Variable importance in projection (VIP) of each PLSR model across data source: tower-based (left column), Ground_VISNIR_ (middle column), and Ground_Fullrange_ models (right column); and predicted parameter: stomatal conductance (top row), predawn leaf water potential (middle row), and midday leaf water potential (bottom row). Fig. S8. Comparison of the tower and handheld instrument PLSR model VIP from Fig. 4 for (A) stomatal conductance, (B) predawn water potential, and (C) midday water potential.
