# Supplementary material for: Hyperspectral Remote Sensing for Phenotyping the Physiological Drought Response of Common and Tepary Bean
Source: Plant Phenomics. 2023 Jan 16;5:0021. doi: 10.34133/plantphenomics.0021 (PMC10076057; doi:10.34133/plantphenomics.0021)
Supplement: Supplementary Materials — Fig. S1. Genotype-specific relative soil moisture content at 20-, 40-, 80-, 120-, and 140-cm depths across 3 field campaigns. Fig. S2. Genotype-specific drought response of stomatal conductance from 8, 10, 12, and 14 h across 3 field campaigns. Fig. S3. Genotype-specific drought response of leaf water potential from predawn (6 h) and midday (13 h) across 3 field campaigns. Fig. S4. Genotype-specific drought response of midday drone-based normalized difference vegetation index across 3 field campaigns. Fig. S5. Genotype-specific drought response of midday drone-based canopy temperature across 3 field campaigns. Fig. S6. Genotype-specific drought response of midday drone-based canopy volume across 3 field campaigns. Fig. S7. Variable importance in projection (VIP) of each PLSR model across data source: tower-based (left column), GroundVISNIR (middle column), and GroundFullrange models (right column); and predicted parameter: stomatal conductance (top row), predawn leaf water potential (middle row), and midday leaf water potential (bottom row). Fig. S8. Comparison of the tower and handheld instrument PLSR model VIP from Fig. 4 for (A) stomatal conductance, (B) predawn water potential, and (C) midday water potential. [file plantphenomics.0021.f1.docx]

Supplementary Figures for:

Hyperspectral remote sensing for phenotyping the physiological drought response of common and tepary bean

Christopher YS Wong^1^, Matthew E Gilbert^1^, Marshall A Pierce^1^, Travis A Parker^1^, Antonia Palkovic^1^, Paul Gepts^1^, Troy S Magney^1,*^, Thomas N Buckley^1,*^

^*^ TNB and TSM should be considered joint senior authors

^1^ Department of Plant Sciences, University of California, Davis, Davis, CA, 95616 USA

Corresponding authors: CYSW ([cyswong@ucdavis.edu](mailto:cyswong@ucdavis.edu)), TSM ([tmagney@ucdavis.edu](mailto:tmagney@ucdavis.edu)), TNB ([tnbuckley@ucdavis.edu](mailto:tnbuckley@ucdavis.edu))


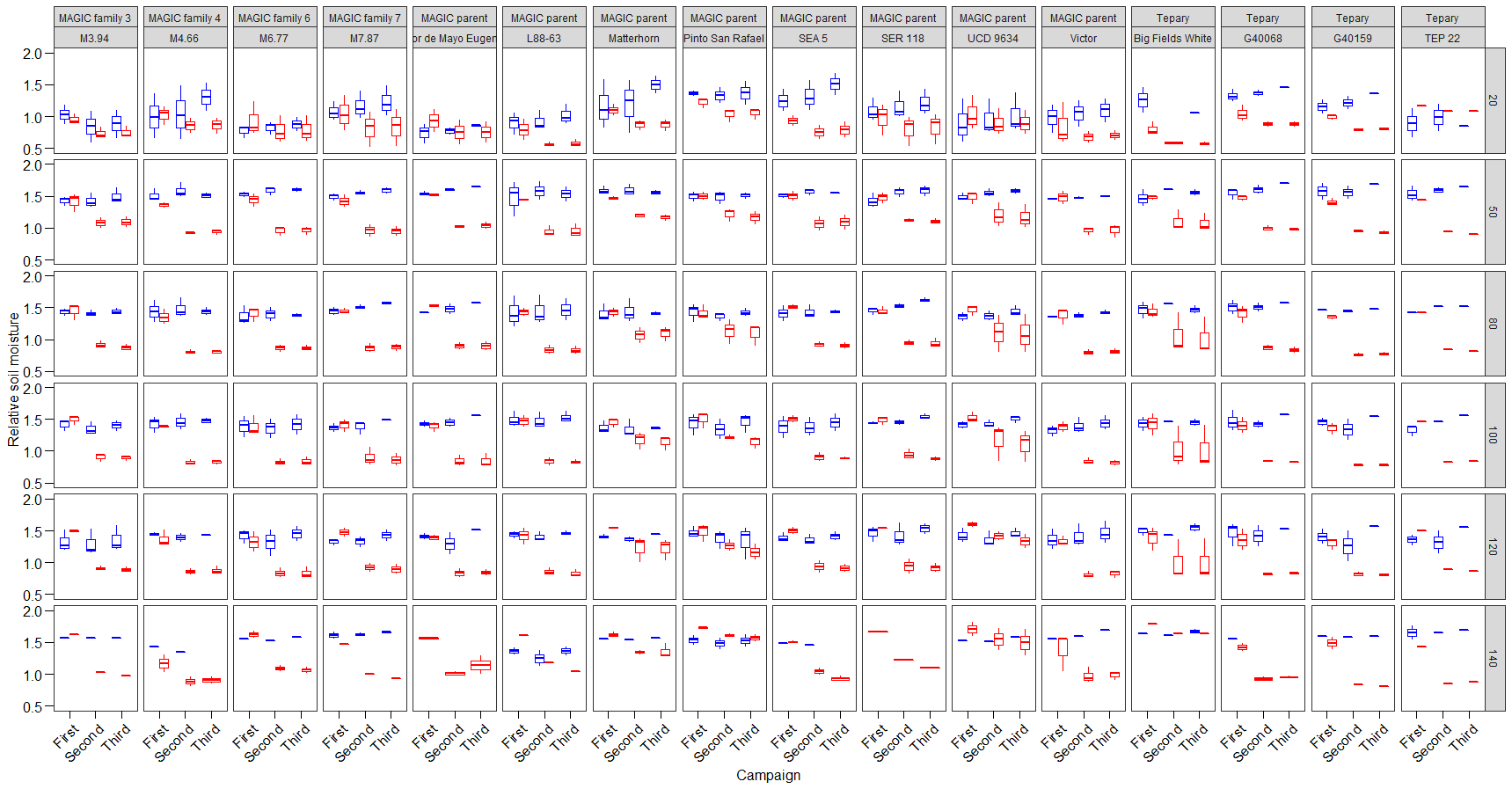


Figure S1. Genotype-specific relative soil moisture content at 20, 40, 80, 120, and 140 cm depths across three field campaigns. Campaigns represent pre-drought baseline (First), and 2 and 4 weeks after terminal drought application (Second and Third, respectively).


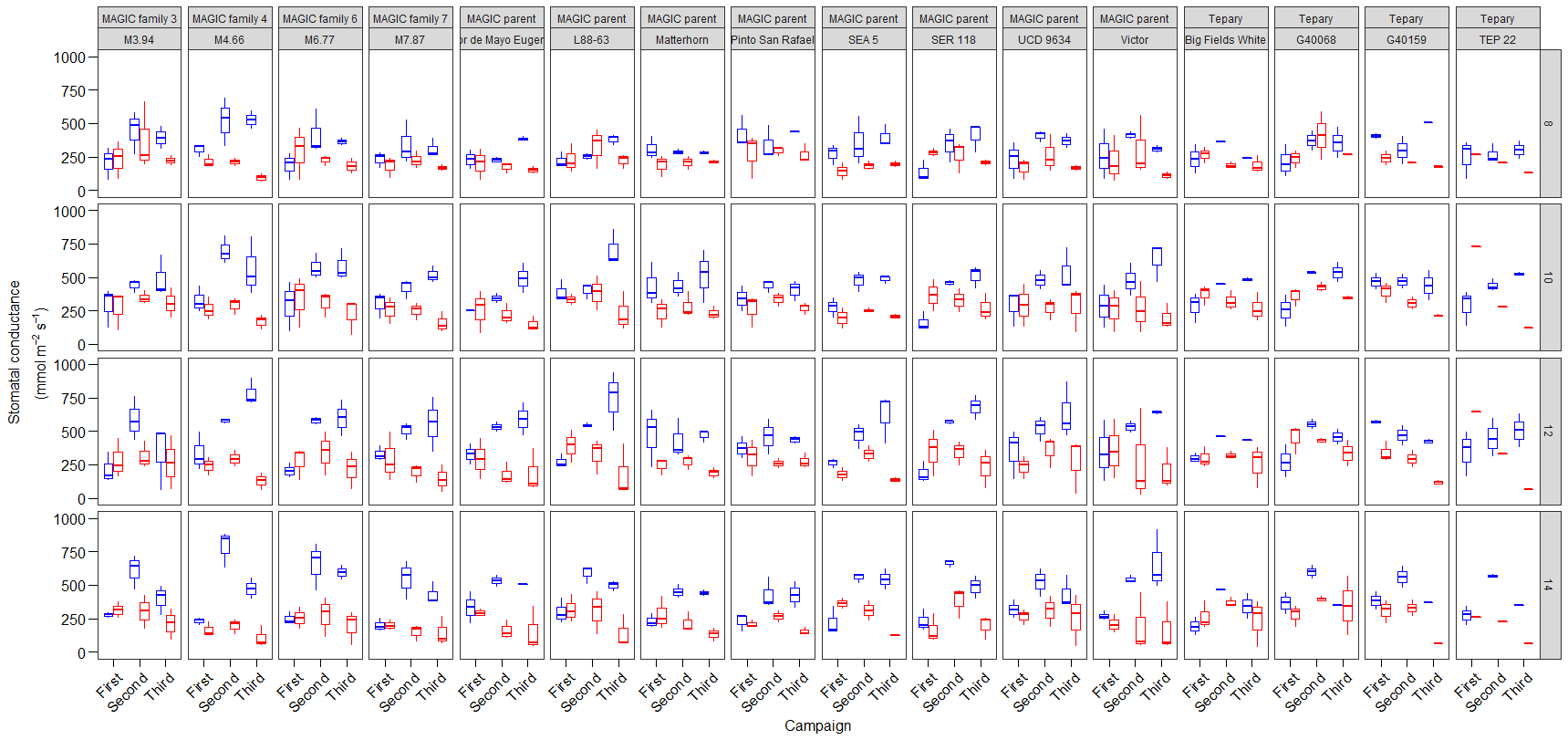


Figure S2. Genotype-specific drought response of stomatal conductance from 8, 10, 12, and 14 h across three field campaigns. Campaigns represent pre-drought baseline (First), and 2 and 4 weeks after terminal drought application (Second and Third, respectively).


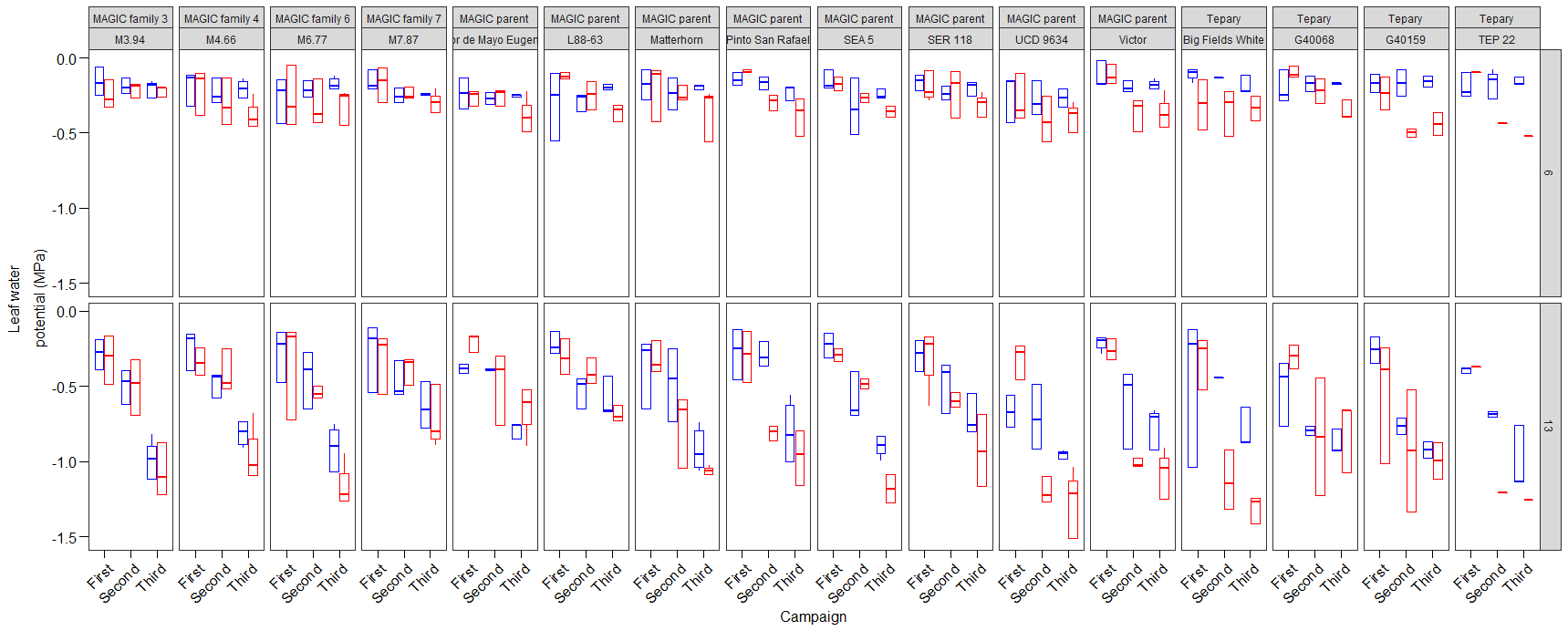


Figure S3. Genotype-specific drought response of leaf water potential from predawn (6 h) and midday (13 h) across three field campaigns. Campaigns represent pre-drought baseline (First), and 2 and 4 weeks after terminal drought application (Second and Third, respectively).


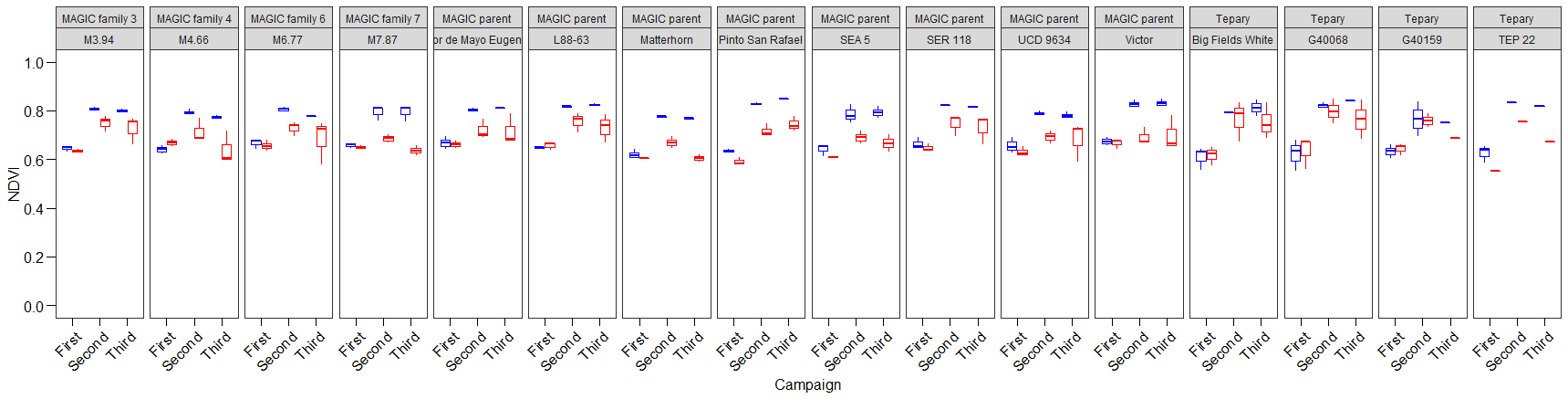


Figure S4. Genotype-specific drought response of midday drone-based normalized difference vegetation index across three field campaigns. Campaigns represent pre-drought baseline (First), and 2 and 4 weeks after terminal drought application (Second and Third, respectively).


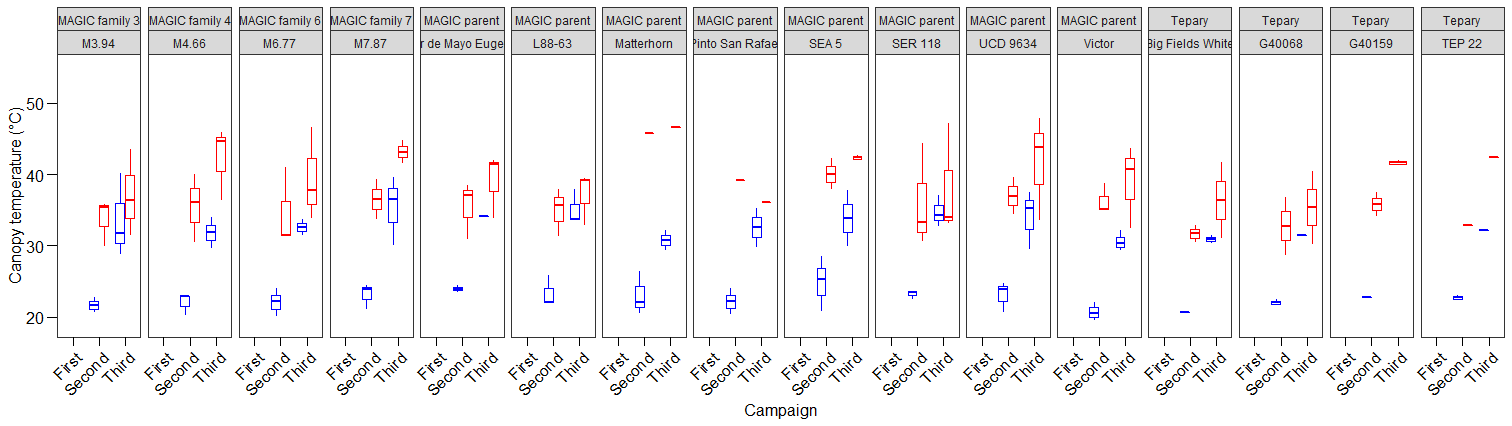


Figure S5. Genotype-specific drought response of midday drone-based canopy temperature across three field campaigns. Campaigns represent pre-drought baseline (First), and 2 and 4 weeks after terminal drought application (Second and Third, respectively).


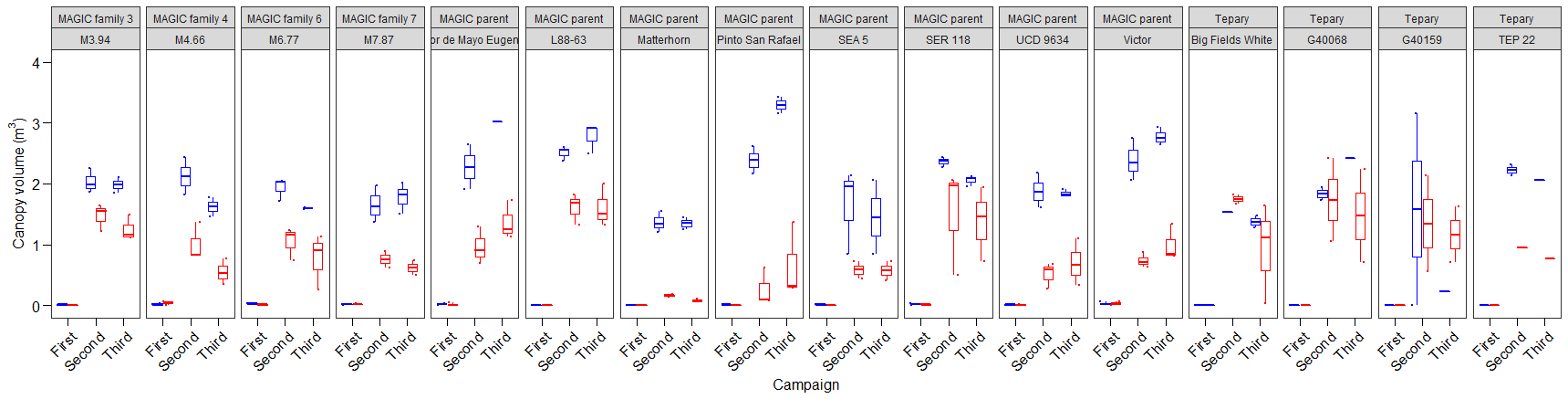


Figure S6. Genotype-specific drought response of midday drone-based canopy volume across three field campaigns. Campaigns represent pre-drought baseline (First), and 2 and 4 weeks after terminal drought application (Second and Third, respectively).


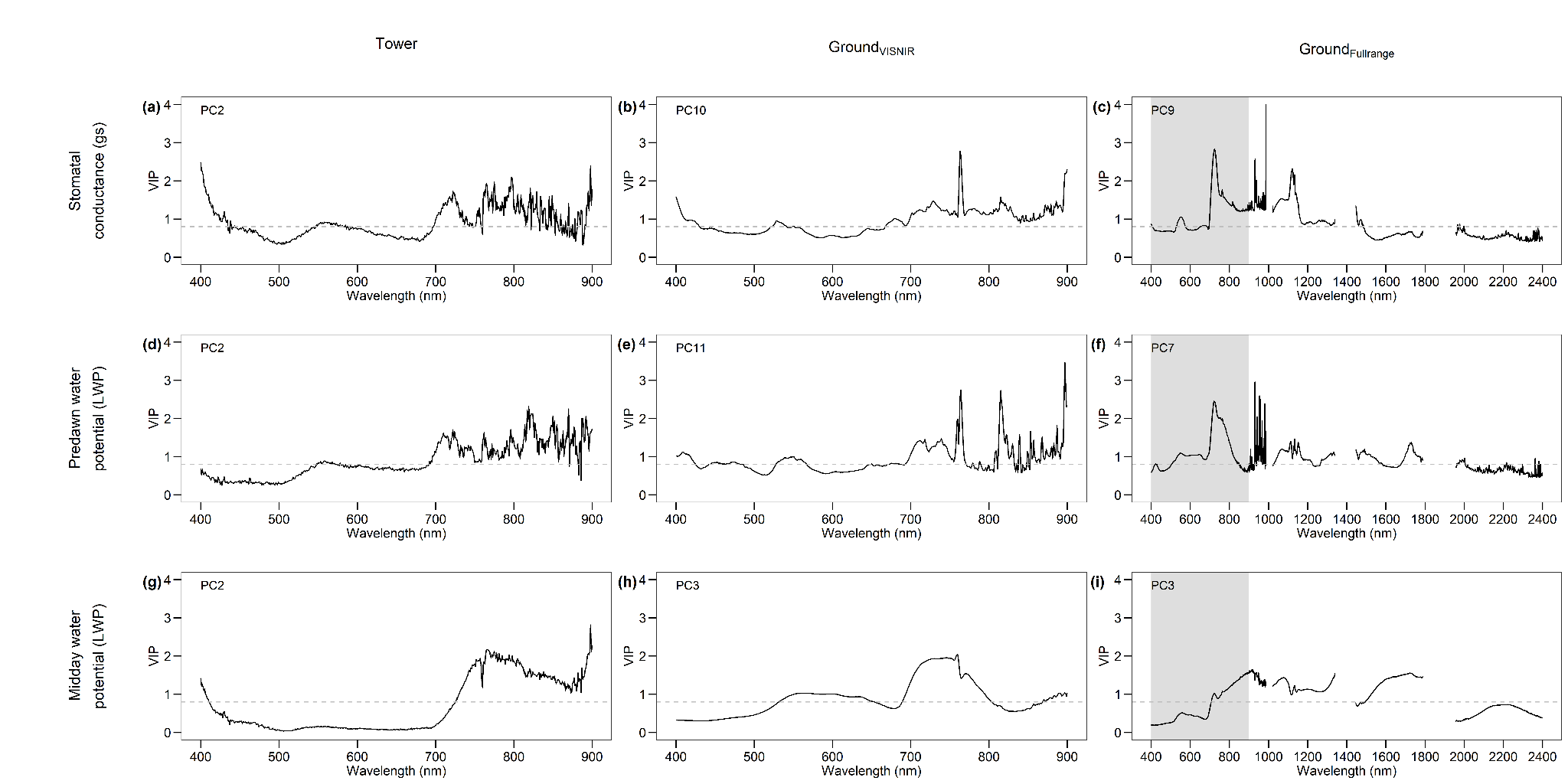


Figure S7. Variable importance in projection (VIP) of each PLSR model across data source: tower-based (left column), Ground_VISNIR_ (middle column), Ground_Fullrange_ models (right column); and predicted parameter: stomatal conductance (top row), predawn leaf water potential (middle row), and midday leaf water potential (bottom row). Gray region shows the 400 to 900 nm range covered by the tower and constrained Ground_VISNIR_ range.


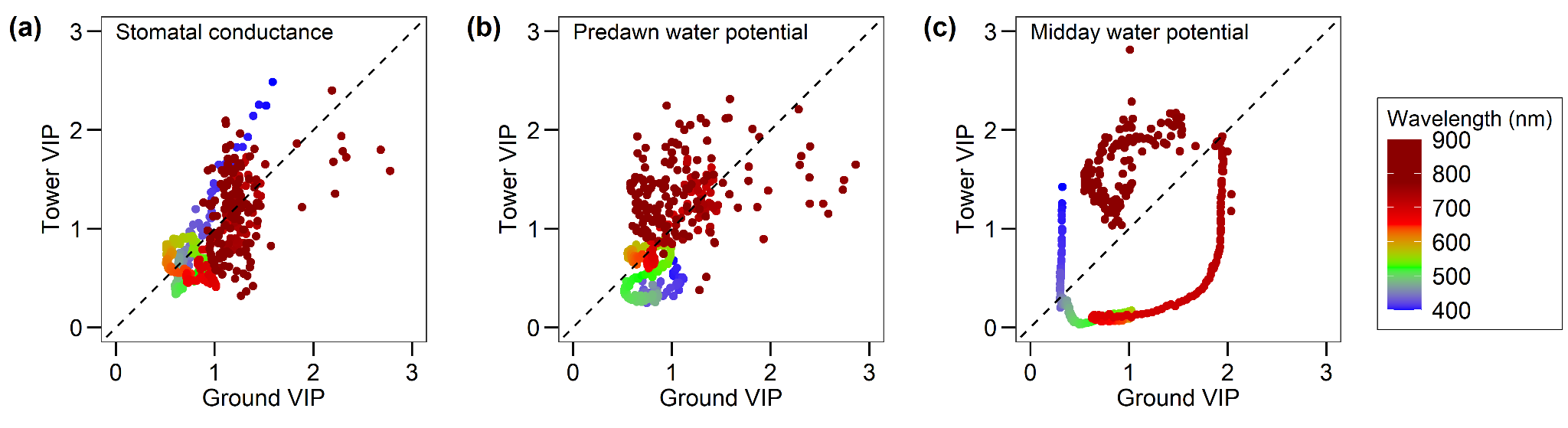


Figure S8. Comparison of the tower and handheld instrument PLSR model variable importance in projection (VIP) from Fig 4 for (a) stomatal conductance, (b) predawn water potential, and (c) midday water potential. Each point represents matching wavebands between scales from Ground_VISNIR_ and tower-based models.
